# Social determinants of health and the prediction of missed breast imaging appointments

**DOI:** 10.1186/s12913-022-08784-8

**Published:** 2022-11-30

**Authors:** Shahabeddin Sotudian, Aaron Afran, Christina A. LeBedis, Anna F. Rives, Ioannis Ch. Paschalidis, Michael D. C. Fishman

**Affiliations:** 1 Department of Electrical and Computer Engineering, Division of Systems Engineering, Boston University, Boston, MA USA; 2 Department of Radiology, Boston University School of Medicine, Boston, MA USA; 3 Department of Radiology, Boston Medical Center, Boston University School of Medicine, Boston, MA USA; 4 Department of Biomedical Engineering, and Faculty of Computing & Data Sciences, Boston University, Boston, MA USA; 5 Rafik B. Hariri Institute for Computing and Computational Science & Engineering, Boston, MA USA

**Keywords:** Social determinants of health, Missed appointment, Predictive model, Radiology, Breast imaging

## Abstract

**Background:**

Predictive models utilizing social determinants of health (SDH), demographic data, and local weather data were trained to predict missed imaging appointments (MIA) among breast imaging patients at the Boston Medical Center (BMC). Patients were characterized by many different variables, including social needs, demographics, imaging utilization, appointment features, and weather conditions on the date of the appointment.

**Methods:**

This HIPAA compliant retrospective cohort study was IRB approved. Informed consent was waived. After data preprocessing steps, the dataset contained 9,970 patients and 36,606 appointments from 1/1/2015 to 12/31/2019. We identified 57 potentially impactful variables used in the initial prediction model and assessed each patient for MIA. We then developed a parsimonious model via recursive feature elimination, which identified the 25 most predictive variables. We utilized linear and non-linear models including support vector machines (SVM), logistic regression (LR), and random forest (RF) to predict MIA and compared their performance.

**Results:**

The highest-performing full model is the nonlinear RF, achieving the highest Area Under the ROC Curve (AUC) of 76% and average F1 score of 85%. Models limited to the most predictive variables were able to attain AUC and F1 scores comparable to models with all variables included. The variables most predictive of missed appointments included timing, prior appointment history, referral department of origin, and socioeconomic factors such as household income and access to caregiving services.

**Conclusions:**

Prediction of MIA with the data available is inherently limited by the complex, multifactorial nature of MIA. However, the algorithms presented achieved acceptable performance and demonstrated that socioeconomic factors were useful predictors of MIA. In contrast with non-modifiable demographic factors, we can address SDH to decrease the incidence of MIA.

**Supplementary Information:**

The online version contains supplementary material available at 10.1186/s12913-022-08784-8.

## Background

Missed imaging appointments (MIA) are a challenge for patients and providers, causing delayed care and inefficiencies. Variables including race, age, sex, housing, and health insurance may be associated with MIA [1, 2]. Screening and diagnostic breast imaging are essential to early detection of breast cancer, which significantly impacts survival [3]. MIA are also inefficient and costly: an average academic medical center in the US loses $1 million annually in potential revenue from missed appointments [4]. Additionally, patient care may suffer due to missed appointments [5–7].

Outside of radiology, several machine learning algorithms have been used for predicting MIA, including the naive Bayes classifier [8], decision tree models [7, 9], artificial neural networks [8], time-frequency analysis models [10], metaheuristic-based models [11], and the most widely used, logistic regression models (LR) [8, 12–14]. In radiology, Harvey et al. [15] developed descriptive statistics and LR models to predict failure to attend a scheduled radiology appointment, achieving 75.3% AUC. Daye et al. [2] assessed the relationship between wait days (WDs) on missed outpatient MRI with multivariate LR and linear regression. In that study, WDs were defined as the time interval between the date that the appointment was made and the actual date of the appointment. They concluded that increased WDs for MRI appointments substantially increases the probability of missed appointments. The pre-appointment likelihood of no-show was predicted by Mieloszyk et al. [16] using LR models, achieving 77% AUC. Lead time was the most impactful feature of their model.

Social determinants of health (SDH) are social and demographic patient characteristics which influence health outcomes [17, 18]. Clearly many other factors affect health; however, they are outweighed by the impact of social and economic factors [19]. Several studies have identified risk factors and variables associated with no-shows and cancellations [11, 20–23]. Thus, SDH should be included in models that attempt to predict imaging appointment outcomes. Boston Medical Center (BMC) has implemented a novel screening & referral tool/program (THRIVE), seeking to understand patient social needs through. THRIVE is a custom SDH screening program that surveys patients on their unmet social needs in eight different domains: transportation insecurity, difficulty arranging child/elder care, ability to pay for utilities, difficulty accessing education resources, food insecurity, housing insecurity, employment insecurity, and difficulty paying for medication [24]. THRIVE, which is not an acronym, allows clinicians to understand and address patients’ unmet social needs.

Machine learning techniques with simple demographic factors can be useful to better understand no-show appointments, both in radiology and other specialties. We aimed to build upon that foundation by including modifiable and potentially more impactful SDH in our analysis. The goal of our study was to create models utilizing SDH, demographics, and environmental data and employ them to predict MIA among breast imaging patients at BMC.

## Methods

### Data description

This HIPAA compliant retrospective study was IRB approved. Informed consent was waived. Our dataset contains 11,296 patients and 53,326 appointments from 1/1/2015 to 12/31/2019. The dataset was de-identified for analysis. Following the removal of duplicate appointments and patients without SDH data, our data set included 36,606 appointments and 9,970 unique patients. Study data includes appointment records, demographic data, and SDH responses obtained from BMC’s electronic health records (EHR) at the research clinical data warehouse.

### Data pre-processing and variable preparation

Demographic variables included age, gender, race/ethnicity, birthplace, primary language, education level, marital status, primary insurance, and estimated address (ZIP Code). Appointment records included reservation date, appointment date, department name, and type of order (e.g., diagnostic mammography, MRI breast biopsy). Using information in appointment records, we created new variables to consider in our analysis. The complete lists of categorical and numerical variables and their characteristics are presented in Tables 1, 2 and 3. Please note that we used all 36,606 appointments to calculate the values in Tables 2 and 3. In these tables, N (%) refers to the percentage of appointments within each category. Attended (%) and Missed (%) denote the percentages of attended and missed appointments for each category. In Table 3, Mean ($\underline{X}$), Q1, Median, and Q3 denote the average, first quartile, second quartile (i.e., median), and third quartile of the corresponding variable, respectively. For each variable, $\underline{X}_{U}\ \left(\underline{X}_{L}\right)$ refers to the set of appointments for which the given variable is above (below) its respective mean ($\underline{X}$).

**Table 1:** Generated variables and their descriptions

Generated variables	Description
Distance	The distance in miles between patient home address and clinic ZIP codes.
Median household income	Annual income was extrapolated from the median household income in each patient’s ZIP code.
Appointment After Long Weekend	Variable indicating if day before the appointment was public holiday.
Cancelation Last Appointment	Variable indicating if the patient’s last appointment was a cancelation.
Waiting Time	The number of days between the date appointment was made and the actual appointment date.
Time Since Prior Appointment	Number of days between current appointment and most recent prior appointment.
Time Since Prior Cancelation	Number of days between current appointment and most recent prior cancelation.
Temperature	Average of hourly temperature on appointment date (Fahrenheit).
Precipitation Intensity	Intensity of precipitation occurring at appointment time (inches of liquid water per hour).
Patient’s appointment history	Features related to patient’s appointment history include total appointments, total cancelations, and total appointments completed.

**Table 2:** Descriptive statistics of the study sample (categorical variables).

Variables			N (%)	Attended (%)	Missed (%)
Primary Race	Black / African American		54.3	72.2	27.8
	White		17.9	72.8	27.2
	Asian		3.2	74.9	25.1
	Hispanic or Latino		11.5	74.1	25.9
	American Indian/Native American		0.4	70.1	29.9
	Native Hawaiian/Pacific Islander		0.1	79.2	20.8
	Declined to report		12.6	76.1	23.9
Language	English		64.2	72.2	27.8
	Spanish		12.9	76.6	23.4
	Other		22.9	73.7	26.3
Education Level	No more than high school		77.6	73.2	26.8
	College or graduate level		22.4	72.6	27.4
Hispanic Indicator	Non-Hispanic		82.1	72.8	27.2
	Hispanic		17.9	74.5	25.5
Marital Status	Single		47.7	72.3	27.7
	Married		34.5	74.6	25.4
	Divorced		7.7	71.9	28.1
	Widowed		5.6	73.1	26.9
	Legally Separated		4.4	72.7	27.3
	Significant Other		0.1	60.0	40.0
Primary Insurance	PRIVATE		47.5	74.1	25.9
	MEDICAID		31.4	71.1	28.9
	MEDICARE		21.1	73.9	26.1
Order Name	Screening		70.4	69.4	30.6
	Diagnostic		25.1	81.2	18.8
	Biopsy		4.5	86.3	13.7
Department Name	Radiology		59.7	79.9	20.1
	Primary Care		30.1	57.5	42.5
	Community Health Centers (CHC)		1.8	93.5	6.5
	Other		8.4	75.9	24.1
Weekday	Monday		18.9	70.4	29.6
	Tuesday		20.8	72.6	27.4
	Wednesday		22.1	73.1	26.9
	Thursday		19.7	73.9	26.1
	Friday		18.5	75.2	24.8
Time	Before 8 A.M.		1.7	52.8	47.2
	8 A.M. to 10 A.M.		25.5	72.3	27.7
	10 A.M. to 12 P.M.		25.6	71.5	28.5
	12 P.M. to 14 P.M.		15.6	72.8	27.2
	14 P.M. to 16 P.M.		20.5	73.4	26.6
	After 16 P.M.		11.1	82.5	17.5
Season	Spring		22.5	70.5	29.5
	Summer		27.2	72.9	27.1
	Fall		29.2	74.5	25.5
	Winter		21.1	74.1	25.9
Appointment After Long Weekend	No		79.3	73.1	26.9
	Yes		20.7	73.4	26.6
Cancelation Last Appointment	No		76.7	72.7	27.3
	Yes		23.3	74.4	25.6
Social Determinants of Health	Housing	No	95.3	73.3	26.7
		Yes	4.7	68.4	31.6
	Food	No	85.9	73.6	26.4
		Yes	14.1	69.7	30.3
	Medications	No	92.1	73.5	26.5
		Yes	7.9	68.9	31.1
	Transportation	No	93.2	73.5	26.5
		Yes	6.8	67.4	32.6
	Utilities	No	90.8	73.5	26.5
		Yes	9.2	68.9	31.1
	Caregiving	No	97.2	73.2	26.8
		Yes	2.8	70.1	29.9
	Employment	No	93.7	73.3	26.7
		Yes	6.3	69.5	30.5
	Education	No	89.1	73.4	26.6
		Yes	10.9	70.9	29.1

**Table 3:** Descriptive statistics of the study sample (numerical variables)

Variables	Q1	Median	Q3	Mean ($\underline{X})$		N(%)	Attended(%)	Missed(%)
Age	51	58	66	58.47	$\underline{X}_{U}$	49.8	74.3	25.7
					$\underline{X}_{L}$	50.2	71.9	28.1
Waiting Time (days)	8	21	36	33.32	$\underline{X}_{U}$	27.6	66.9	33.1
					$\underline{X}_{L}$	72.4	75.4	24.6
Total Appointments	1	1	3	1.92	$\underline{X}_{U}$	49.1	75.2	24.8
					$\underline{X}_{L}$	50.8	70.9	29.1
Total Cancellations	0	0	1	0.62	$\underline{X}_{U}$	41.2	73.3	26.7
					$\underline{X}_{L}$	58.8	72.9	27.1
Total Completed Appointments	0	1	2	1.31	$\underline{X}_{U}$	34.5	77.4	22.6
					$\underline{X}_{L}$	65.5	70.8	29.2
Time Since Prior Appointment (days)	Less than a day	28	370	173.84	$\underline{X}_{U}$	38.4	66.9	33.1
					$\underline{X}_{L}$	61.6	76.9	23.1
Time Since Prior Cancellation (days)	Less than a day	1	47	96.87	$\underline{X}_{U}$	21.4	64.3	35.7
					$\underline{X}_{L}$	78.6	75.5	24.5
Temperature (°***F***)	31.27	47.32	62.20	46.08	$\underline{X}_{U}$	51.8	73.3	26.7
					$\underline{X}_{L}$	48.2	72.9	27.1
Precipitation Intensity (in/h)	0	0.0003	0.003	0.0043	$\underline{X}_{U}$	22.5	72.9	27.1
					$\underline{X}_{L}$	77.5	73.7	26.3
Distance (miles)	3.43	6.67	12.16	11.41	$\underline{X}_{U}$	22.1	72.9	27.1
					$\underline{X}_{L}$	77.9	73.1	26.9
Median Household Income (USD)	45130	51863	66735	55302.32	$\underline{X}_{U}$	40.9	73.2	26.8
					$\underline{X}_{L}$	59.1	73.1	26.9

As will be discussed later, we ultimately want to use the last appointment of each patient (i.e., 9,970 samples) to develop our predictive models. We have three types of appointments (i.e., "Order Name" in Table 2), namely diagnostic, biopsy, and screening. Approximately 96% of appointments are diagnostic or screening. All 9,970 patients have at least one screening appointment. In our work, the term “imaging appointments” refers to both diagnostic and screening appointments. Some categorical variables such as time, type of order, and primary insurance were categorized into fewer categories to simplify analysis. As for SDH variables, we created eight indicator variables for each of the eight domains. The value of a domain will be encoded as ‘1’ If a patient reports the corresponding social need. Otherwise, if the answer is ‘No need’ or the answer is missing, the value is set to ‘0’. The SDH information was obtained using THRIVE, which was integrated into the BMC's EHR starting in 2017. THRIVE is based on a short questionnaire, which is voluntarily completed by patients at each clinic visit. The THRIVE questionnaire and the summary of THRIVE data pre-processing steps are provided in the Supplement (see Additional file 1).

For SDH variables we provide the percentages of those who answered Yes/No to the question of having the specific need and the corresponding percentages of attended/missed appointments for each cohort.

Most of the features in our data set do not contain any missing values except “Marital Status”, “Education Level”, “Hispanic Indicator”, and “Primary Race”. We used the mode of each feature to impute the existing missing values. Table 4 presents the number of missing values for each feature and the mode of feature that we used for imputation.

**Table 4:** Missing values in the data set

Feature name	Number of missing (%)	Mode
Marital Status	131 (1.3%)	‘Single’
Education Level	687 (6.9%)	‘No more than high school’
Hispanic Indicator	10 (0.1%)	‘Non-Hispanic’
Primary Race	8 (0.08 %)	‘Black / African American’

Continuous variables were scaled to lie between zero and one. To mitigate the effect of outliers, we substituted each variable with values higher than the 99th percentile or lower than the 1st percentile with the 99th or 1st percentile, respectively. Categorical variables such as primary race and education level were converted to numerical by ‘one-hot’ encoding. Each categorical variable was encoded as an indicator variable for each category, yielding 57 variables for each patient.

To reduce the dimensionality of the data and find the most informative features for our model, we used an *l*_1_-norm regularized Support Vector Machine algorithm (SVM-L1) for recursive feature elimination. Several studies [25, 26] demonstrated the effectiveness of feature selection via regularization in biomedical applications.

Linear models like SVM penalized with the *l*_1_ norm induce sparse solutions. In SVM, the parameter C (a.k.a. the soft margin constant or misclassification penalty) controls the sparsity of the model where the smaller C the fewer features selected, since this has the effect of increasing the importance of the *l*_1_-norm regularizer. We used recursive feature elimination as follows. We started with all features and progressively dropped less informative features (i.e., a feature that has minimal absolute coefficient) by decreasing the parameter C [27]. The Supplement includes additional details (see Additional file 1). This method selected 20 features for our final model. The complete list of these features will be presented shortly. There were three SDH variables (i.e., housing, transportation, and utilities) between these 20 features. Since we wanted to examine the effect of the SDH variables on MIA, we manually added the other five SDH variables to our final selected features. Thus, we used 25 features in our parsimonious model.

### Classification methods

We employed non-linear and linear classifiers including random forest (RF) [28], XGBoost [29], and the regularized versions of support vector machine (SVM) [30] and logistic regression (LR) [31] using *l*_1_ or *l*_2_-norm regularization.

The random forest (RF) is an ensemble algorithm that combines the prediction of multiple decision tree classifiers [28]. RF trains multiple decision trees in parallel using a random subset of the training set and features. The trained classifiers are used to classify a test sample and all classifiers are combined by majority voting. Combining multiple decision trees and RF randomness prevents overfitting and reduces model variance.

XGBoost is an ensemble tree algorithm [29]; it generates a large number of decision trees in sequential order so the training samples misclassified by the previous tree receive a higher weight. This process repeats until the number of trees reaches a predetermined number. Eventually, all trained trees are weighted together to produce a final decision. Shrinkage and column subsampling in XGBoost prevent overfitting [32].

RF and XGBoost are non-linear algorithms that are difficult to interpret (often involving hundreds of decision trees) but are useful because they may indicate what is the best classification performance one could obtain. We also employed custom linear classifiers, including the support vector machine (SVM) [30] and logistic regression (LR) [31] which can yield interpretable models. SVM constructs a hyperplane that separates the two classes to maximize the margin between samples while minimizing misclassification errors. We used the linear SVM, but the method can be extended to allow for non-linear decision surfaces.

LR is a regression algorithm for predicting a dichotomous dependent variable. It uses a linear regression model to approximate the logarithm of the odds of the dependent variable (outcome) [33]. The regularized versions of LR and SVM (using *l*_1_ or *l*_2_-norm regularization) were considered to improve the robustness of these algorithms in the presence of noise and outliers [31]. We used open-source python packages (i.e., Scikit-learn [34] and Statsmodels [35]) to implement our predictive models.

### Performance metrics

The predictive models were assessed using three performance metrics, namely Area Under the Curve (AUC, a.k.a. C-statistic) of the Receiver Operating Characteristic (ROC), the Micro-F1 score, and the Weighted-F1 score. The ROC plots sensitivity (or recall) against one minus the specificity. Values are between 0 and 1 with a higher AUC value indicating better predictive capability of the model. The F1 score is defined as the harmonic mean of recall and precision. Precision refers to the number of appointments in the real positive class (e.g., truly missed appointments) over the number of appointments predicted in the positive class. The Micro-averaged F1 score aggregates the contributions of both classes to compute the harmonic mean while the Weighted-F1 score is calculated by weighting the F1-score of each class by the number of appointments in that class.

### Outcome and experimental settings

Our primary outcome (class 1) is MIA which can be defined as any scheduled imaging appointment not performed, canceled, or rescheduled before the scheduled time. Since the later appointments of a patient contain past information (e.g., total cancellations, total appointments, and so on), we cannot assume appointments for the same patients are independent of each other. Since independence is needed for training predictive models, we only used the last appointment of each patient in our predictive models. Thus, in total, we used 9,970 appointments for model development and validation.

The data were split into a training (80%) and a test set (20%). Algorithm parameters were optimized on the training (derivation) set using five-fold cross-validation. Performance metrics were computed on the test set. This process was repeated five times, each time with a random split into training/testing sets to ensure the robustness of our results. Please note that we optimized algorithm parameters to maximize AUC. The average and standard deviation of performance metrics on the test set over the 5 random splits are presented.

We considered three sets of features for these models. All 57 features were used to develop “full models.” Using the feature selection procedure outlined in Section 2.2 (i.e., SVM-L1 feature selection method**)**, we developed parsimonious models with the most impactful variables. To show the impact of the SVM-L1 feature selection method, we also reported the result of a Univariate Feature Selection (UFS) method. To that end, we used a chi-squared test to select the twenty-five best features. In feature selection, the goal is to select features that are highly dependent on the output. When two features are independent, the observed count is close to the expected count. Therefore, we observe a smaller chi-squared value. In other words, we select a feature for model development if the feature is more dependent on the output which means a higher chi-squared value. Interestingly, twenty features out of 25 UFS selected features are the same as features that we selected using our SVM-L1 method. Specifically, UFS selected “Order name – diagnostic,” “Time - 8 A.M. to 10 A.M.,” “Weekday – Monday,” “Days Since Last Appointment,” and “Primary Insurance - Medicare” instead of “Median Household Income,” “Distance,” “Primary Insurance – Private,” “SDH – Caregiving,” and “Temperature”. The other 20 features are the same.

Here, the coefficients of the LR parsimonious model are of great importance. After standardizing the variables, a larger absolute coefficient suggests that the likelihood is more sensitive to this specific variable [31]. The sign of the coefficient indicates the direction of correlation with MIA. We also used the odds ratio (OR) and marginal effect (ME) [36] in our analysis. The odds ratio (OR) represents the odds that MIA will occur (probability *p* it will occur over 1 − *p*) given a particular binary variable divided by the odds of MIA in the absence of that variable. For continuous variables, the odds ratio corresponds to the ratio of the odds induced by a unit increase in the respective variable. An OR greater than one implies that a variable increases the odds of MIA. Marginal effect can be described as the change in the predicted probability of a binary outcome as the risk factor changes by 1 unit holding all other variables in the model constant [36]. Marginal effect provides a simpler way to compare the relative importance of various features in the model and quantify the incremental risk associated with each factor. In logistic regression, we cannot define a single marginal effect for all samples. The most common way is to report the average marginal effect across all samples in the data set [36].

## Results

### Model performance

From the 9,970 appointments included in the study, there are 1,381 MIA, and 8,589 non-MIA. Therefore, the overall proportion of MIA was 13.8%, considerably higher than that of Harvey et al. [15] which included many different radiology modalities and reported a no-show rate of 6.5%. It should be noted that 13.8% is the percentage of missed appointments if we just consider the last appointment of each patient (i.e., 9,970 appointments). Here, we present the results of two types of models for MIA prediction, namely linear (SVM and LR) and non-linear (RF and XGBoost) models. Moreover, we consider three sets of features for these models. All 57 features were used to develop “full models.” These models utilize many variables, making them challenging to interpret. We then developed two parsimonious versions of these models using the feature selection techniques, namely SVM-L1 and UFS. The parsimonious models have 25 features, including all 8 SDH variables. Models with fewer features have several advantages; they (i) are easier to interpret, (ii) require less training time, (iii) have less data redundancy, and (iv) are easier to implement in a clinical setting.

We utilized linear and non-linear models to predict MIA among scheduled radiology appointments at BMC. The highest-performance model (using all 57 features) was the RF that achieved an average AUC of 76% and an average F1 score of 85%. However, similar performance was achieved with a parsimonious model utilizing just 25 variables as well as linear models. Particularly, RF was the best parsimonious model with SVM-L1 features. It has a similar AUC of 75.7%, as high as the model with all 57 features. The same model achieved an average F1 score of 84.2% that is slightly less than the performance of the full model. Moreover, the performance of parsimonious models using SVM-L1 features is better than the models trained using the UFS features. This analysis clearly shows the advantages of sophisticated feature selection methods like SVM-L1.

Table 5 presents the predictive model performance of the full and parsimonious models. Table 5 also lists the 25 variables in the LR-L2 parsimonious model, the LR coefficients of each variable (*Coef*), the correlation of the variable with the outcome (*Y*_*corr*_), the mean of the variable ($\underline{X}_{1}$) for MIA, and the mean of the variable ($\underline{X}_{0}$) for attended appointments. ORs are reported with their 95% confidence intervals (OR 95% CI). Table 5 also presents Marginal Effects (ME) and p-values. The values inside the parentheses refer to the standard deviation of the corresponding metric. SVM-L2 and LR-L2 refer to the *l*_2_-norm regularized SVM and LR models. Note that the coefficients listed for each variable are from the LR-L2 model. We also presented the feature importance of XGBoost in the Supplement (see Additional file 1).

**Table 5:** MIA prediction models: Performance metrics of full and parsimonious models.

	**AUC**		**F1-micro**			**F1-weighted**			
	**Full Models using all 57 features**								
SVM-L2	72.9% (1.5%)		83.4% (1.0%)			81.1% (0.9%)			
LR-L2	72.9% (1.6%)		83.2% (0.4%)			81.3% (1.0%)			
XGBoost	75.1% (1.0%)		84.4% (0.8%)			82.4% (0.9%)			
RF	75.7% (0.6%)		84.9% (1.2%)			82.5% (0.7%)			
	**Parsimonious Models using 25 features (SVM-L1)**								
SVM-L2	72.9% (1.4%)		83.4% (1.0%)			81.1% (0.9%)			
LR-L2	72.8% (1.4%)		83.4% (1.1%)			81.1% (1.1%)			
XGBoost	75.5% (1.1%)		83.9% (1.7%)			82.1% (0.8%)			
RF	75.7% (1.3%)		84.2% (0.9%)			82.3% (0.7%)			
	**Parsimonious Models using 25 features (UFS)**								
SVM-L2	72.5% (1.7%)		82.8% (0.8%)				81.0% (1.0%)		
LR-L2	72.6% (1.7%)		82.7% (1.0%)				80.9% (1.0%)		
XGBoost	75.1% (0.8%)		84.3% (1.8%)				82.2% (0.8%)		
RF	75.2% (0.9%)		83.6% (2.0%)				82.3% (0.9%)		
**Variable**	**Coef**	**ME**	**OR**	**OR 95% CI**		*X*_1_	*X*_0_	*Y*_*corr*_	***P*-Value**
Language - Spanish	-0.231	-0.0237	0.794	0.653	0.964	0.104	0.127	-0.024	0.014
Time Since Prior Cancellation	0.946	0.0001	1.001	1.0005	1.001	224.021	161.099	0.079	<0.001
Season - Spring	-0.296	-0.0303	0.744	0.629	0.880	0.184	0.218	-0.029	<0.001
Primary Insurance - Private	-0.194	-0.0211	0.824	0.729	0.931	0.529	0.550	-0.015	0.002
Department – Community Health Center	-1.926	-0.1152	0.146	0.058	0.364	0.004	0.020	-0.042	<0.001
Department – Primary Care	0.389	0.0425	1.476	1.199	1.816	0.648	0.295	0.256	<0.001
Season - Winter	-0.343	-0.0350	0.710	0.579	0.870	0.188	0.231	-0.035	<0.001
Order Name - Diagnostic	-0.161	-0.0178	0.851	0.728	0.995	0.752	0.749	0.002	0.047
Cancelation Last Appointment	0.070	0.0077	1.073	0.931	1.237	0.267	0.270	-0.002	0.336
Marital Status - Married	-0.183	-0.0194	0.833	0.730	0.951	0.294	0.349	-0.040	0.006
Time - After 16 P.M.	0.006	0.0007	1.006	0.811	1.249	0.084	0.095	-0.014	0.955
Total Completed Appointments	-0.862	-0.0055	0.951	0.912	0.991	1.489	1.826	-0.072	0.016
Department – Radiology	-1.359	-0.1443	0.257	0.207	0.320	0.226	0.600	-0.259	<0.001
Time - Before 8 A.M.	0.838	0.1123	2.312	1.434	3.727	0.015	0.018	-0.007	0.004
Temperature	-0.297	-0.0003	0.997	0.993	1.001	46.050	44.831	0.021	0.142
Median Household Income	-0.650	-0.0000004	0.999	0.999	0.999	54383	55619	-0.021	0.028
Distance	0.100	0.0001	1.001	0.996	1.005	11.499	11.497	0.000	0.763
SDH - Housing	0.310	0.0363	1.364	1.051	1.770	0.069	0.047	0.035	0.030
SDH - Transportation	0.211	0.0240	1.235	0.966	1.577	0.095	0.067	0.037	0.109
SDH - Utilities	0.248	0.0284	1.282	1.021	1.609	0.111	0.086	0.031	0.043
SDH – Food	-0.018	-0.0020	0.982	0.799	1.205	0.163	0.137	0.025	0.859
SDH - Medications	-0.027	-0.0029	0.973	0.766	1.237	0.097	0.080	0.021	0.824
SDH - Caregiving	-0.670	-0.0595	0.512	0.332	0.789	0.021	0.029	-0.018	<0.001
SDH - Employment	-0.146	-0.0152	0.864	0.660	1.133	0.070	0.065	0.006	0.273
SDH - Education	0.009	0.0010	1.009	0.817	1.247	0.125	0.110	0.016	0.933
Constant Coef	-0.794	-	-	-	-	-	-	-	-

Overall, the most impactful variables (i.e., with higher absolute coefficient values) on missed appointments were characteristics of the appointment, such as the timing of the appointment and source of referral, the patient’s appointment history, and socioeconomic features such as median household income and the SDH ‘Caregiving’ variable, indicating having trouble providing care for children, family members, or friends.

### Appointment features

Appointments before 8 am (OR=2.312, ME=0.112) were more likely to be missed. Patients referred to imaging from Community Health Centers (CHCs) were less likely to miss their appointments (OR=0.146, ME=-0.115). Similarly, patients referred to imaging from the Radiology department were less likely to miss their appointments (OR=0.257, ME=-0.144). In contrast, patients referred from the Primary Care Department at BMC were more likely to miss their appointments (OR=1.476, ME=0.042). Patients with more past appointments completed had fewer MIA (OR=0.951, ME=-0.006). Additionally, longer time intervals following previously cancelled appointments were also associated with more MIA, with the odds of MIA increasing by approximately 1.001 each day elapsed. Appointments occurring in the spring and winter were less likely to be missed with the odds of MIA decreasing by 0.744 and 0.710, respectively. Finally, patients who scheduled a diagnostic appointment were less likely to miss their appointments (OR=0.851, ME=-0.018).

### Demographics

Patients whose primary language is Spanish had fewer MIA (OR=0.794, ME=-0.024). Patients with private insurance were less likely to miss appointments than those with Medicare or Medicaid (OR=0.824, ME=-0.021). Married individuals were less likely to miss appointments (OR=0.833, ME=-0.019).

### Socioeconomic factors

Three SDH studied were observed to have a statistically significant impact on MIA, namely housing insecurity, difficulty paying utility bills and caretaking. Patients who are at risk of becoming homeless (OR=1.364, ME=0.036) or have trouble paying utility bills (OR=1.282, ME=0.028) were more likely to miss their appointments. On the other hand, patients who had trouble taking care of a child, family member or friend had fewer missed appointments (OR=0.512, ME=-0.059). Moreover, higher income was associated with fewer MIA (OR=0.999, ME=-0.0000004). Furthermore, inadequate access to transportation (OR=1.235, ME=0.024) came close to statistical association with more missed appointments.

## Discussion

Prediction of MIA helps clinicians introducing targeted interventions to efficiently utilize limited imaging capacity, improve radiology scheduling systems, and ultimately increase access to care. Due to their complex and multifactorial etiologies, predicting MIA with high accuracy may not be practical. However, the algorithms presented here achieved acceptable performance and did elucidate useful information about the variables most predictive of MIA.

Notably, appointment timing was shown to be a good indicator of MIA. MIA before 8 am may occur due to unexpected life events interfering (e.g., dropping children off at school), whereas patients may have an easier time attending appointments in the late afternoon after work. Morning rush-hour traffic could also be a contributing factor. Additionally, orders originating in the Primary Care Department at BMC were more likely missed than those referred from Community Health Centers (CHCs) and Radiology. These clinics serve as primary care, referring patients to BMC for breast imaging. Variety in patient populations is less likely to explain this finding, as SDH and demographics were controlled for. It is possible that patients who typically access care locally at CHCs plan carefully for travel to BMC and may schedule appointments with support of navigators onsite at CHCs, leading to fewer MIA. With this information, we can target patients coming from Primary Care with reminders and offer them enhanced patient navigation.

Diagnostic appointments were less likely to be missed. The importance of diagnostic appointments, evaluation of indeterminate clinical exam findings or of inconclusive findings on screening mammogram, may serve as a motivator for patients not to miss their appointment. Patients who kept past appointments tend to remain reliable, which is expected if other factors in their lives remained constant over time. The odds of patients missing an appointment increased as time elapsed from their last appointment, whether or not they attended that appointment. Patients may lose touch with the health system when appointments are spaced over a longer time interval.

The finding of Spanish-speaking patients being observed to have fewer MIA is challenging to explain, as there is no obvious connection between primary language and missed appointments. However, this is consistent with findings that patients requiring interpreters were less likely to miss appointments [8]. This finding remains largely unexplained, as current clinical practice is to call interpreter services on an iPad upon patient arrival.

Patients with private insurance may miss fewer appointments, as they often have greater financial resources than those on public insurance, such as Medicaid. With greater financial resources, it is easier to keep appointments despite unexpected life events. This finding is consistent with findings by Daye et al [2] and Harvey et al. [15] that patients with noncommercial and Medicaid insurance, respectively, had more missed appointments. However, in contrast to these studies, we did not find associations between patient race and MIA. The patient demographics in our study population differs considerably compared to the population in the two other studies, both performed at another academic medical center in our city. Married people may miss fewer appointments due to social and/or financial support from partners, increasing their ability to attend appointments. In addition, partners may be providing assistance with transportation beyond detection by the THRIVE screener for unmet transportation needs.

It is well-documented that socioeconomic factors can negatively impact health outcomes [8, 37]. Housing insecurity and inability to afford utility bills are emblematic of financial strain, which could make MIA more likely given that they could result in uncovered cost-sharing expenses for patients. Patients who care for a dependent family member or friend may plan ahead for travel to medical appointments and may need to hire a caregiver themselves, possibly leading to fewer MIA. Patients with higher median household incomes have greater financial capability that makes it easier to keep appointments. In contrast with non-modifiable demographic factors, we can address SDH to decrease the incidence of MIA. For example, identifying transportation as an impactful SDH would justify providing ride-share vouchers to selected patients.

Prediction of MIA with the data available is inherently limited by the complex, multifactorial nature of MIA, which also are influenced by variables not included in this study. Since the variables associated with MIA cannot be studied in a randomized, controlled trial, it is not appropriate to make causal inferences from these data. It is possible that other unidentified variables are truly responsible for the relationships observed, which may limit the generalizability of our results. Moreover, generalizability of our findings to other settings may be limited due to the unique patient support mechanisms at our institution and the diversity of population, including 54% black patients and over 50% with public insurance. While missed appointments could be prevented by addressing the variables associated with MIA identified by the prediction model, these variables may vary by institution based on patient demographics and insurance payor mix.

The most impactful variables on missed appointments in our breast imaging patient population, included appointment timing, prior appointment history, referral department of origin, and socioeconomic factors such as household income and access to caregiving services. With more complete data on appointment characteristics as well as patient SDH and demographics, it may be possible to achieve better predictive performance. Since this study includes only approximately 15 months of THRIVE data, many patients have not been screened for unmet social needs. Additional data collected in the future may provide more focused insights on how these variables are associated with MIA and potentially strengthen these predictive models.

## Supplementary Information

**Additional file 1.**

## Data Availability

The data that support the findings of this study are available from Boston Medical Center in Massachusetts but restrictions apply to the availability of these data, which were used under license for the current study, and so are not publicly available. Data are however available from the authors upon reasonable request and with permission of Boston Medical Center. The codes for preprocessing, modeling, and evaluation can be found in the following GitHub repository: https://github.com/noc-lab/Social-Determinants-of-Health-and-the-Prediction-of-Missed-Breast-Imaging-Appointments

## Abbreviations

SDH: Social Determinants of Health
MIA: Missed Imaging Appointments
LR: Logistic Regression
RF: Random Forest
SVM: Support Vector Machines
AUC: Area Under the ROC Curve
CHC: Community Health Centers
OR: Odds Ratio
ME: Marginal Effects
BMC: Boston Medical Center
