# Supplementary material for: Social determinants of health and the prediction of missed breast imaging appointments
Source: BMC Health Serv Res. 2022 Nov 30;22:1454. doi: 10.1186/s12913-022-08784-8 (PMC9714014; doi:10.1186/s12913-022-08784-8)
Supplement: Supplementary file 1 — Additional file 1. [file 12913_2022_8784_MOESM1_ESM.docx]

**Social Determinants of Health and the Prediction of Missed Breast Imaging Appointments**

Shahabeddin Sotudian^1^, Aaron Afran ^2^, Christina A. LeBedis^2,3^,

Anna F. Rives^2,3^, Ioannis Ch. Paschalidis^1,4*^, Michael D.C. Fishman^2,3^

^1^Department of Electrical and Computer Engineering, Division of Systems Engineering, Boston University, Boston, MA, United States

^2^Department of Radiology, Boston University School of Medicine, Boston, MA, United States.

^3^Department of Radiology, Boston Medical Center, Boston University School of Medicine, Boston, MA, United States

^4^Department of Biomedical Engineering, and Faculty of Computing & Data Sciences, Boston University, Boston, MA, United States

**Supporting information**

**S1. THRIVE data pre-processing**

Boston Medical Center (BMC) developed a novel, custom Social Determinants of Health (SDH) screening model (a.k.a. THRIVE) with EHR-integration and auto-generated targeted resource referrals based on SDH responses for identifying Health-Related Social Needs (HRSN) among its patient populations. It assesses for HSRN within the following eight domains: Housing, Food, Affordable Medications, Transportation, Utilities, Caregiving, Education, and Employment. We identified patients that screen positive for HRSN on the THRIVE questionnaire and the control group who screened negative. To that end, we generated 8 binary variables using the THRIVE questionnaire as follows:

**Housing Insecurity:** Do you think you are at risk of becoming homeless? What is your living situation today?

The following possible responses were considered as “Yes” for Housing Insecurity:

- "I do not have a steady place to live (I am temporarily staying with others, in a hotel, in a shelter, living outside on the street, on a bench, in a car, abandoned building, bus or train station, or in a park)"
- "I have a place to live today, but I am worried about losing it in the future"
- "Yes"

The following possible responses were considered as “No” for Housing Insecurity:

- "I have a steady place to live"
- “No”

**Food Insecurity:** “Within the last 12 months, were you worried whether your food would run out before you got money to buy more? Is this an emergency, do you need food for tonight?”

The following possible responses were considered as “Yes” for Food Insecurity:

- "Yes"
- “Often true”
- “Sometimes true”

The following responses were considered as “No” for Food Insecurity:

- “No”
- “Never true”

The following survey questions were asked in Yes/No format:

**Medications:** “Do you have trouble paying for your medications?”

**Transportation:** Do you have trouble getting transportation to medical appointments?”

**Utilities:** Do you have trouble paying your heating or electricity bill?

**Caretaking: “**Do you have trouble taking care of a child, family member or friend?”

**Employment:** “Are you currently unemployed and looking for a job?”

**Education:** “Are you interested in more education?”

**S2. Recursive feature elimination using SVM-L1**

We used an $l_{1}$-norm regularized Support Vector Machine algorithm (SVM-L1) for recursive feature elimination. Specifically, we started with all features and progressively removed less informative features (i.e., a feature that has minimal absolute coefficient) by decreasing the parameter C in SVM-L1 algorithm. At each iteration, we computed the AUC of the model with the selected features. To that end, all appointments were split into a training set and a validation set, where 70% of all appointments formed the feature selection training set, and the remaining 30% were assigned to the feature selection validation set and exclusively used for measuring the AUC of the algorithm for different subsets of features. Fig S1 demonstrates the AUC path where x- and y-axes denote the size of the feature subset and the AUC score, respectively. The AUC score plateaued after 20 features (AUC = 0.7021), which were used to train our final model. The complete list of these features can be found in the main manuscript. As we mentioned, there were three SDH variables (i.e., housing, transportation, and utilities) between these 20 features. Since we wanted to examine the effect of the SDH variables on MIA, we manually added the other five SDH variables to our final selected features.


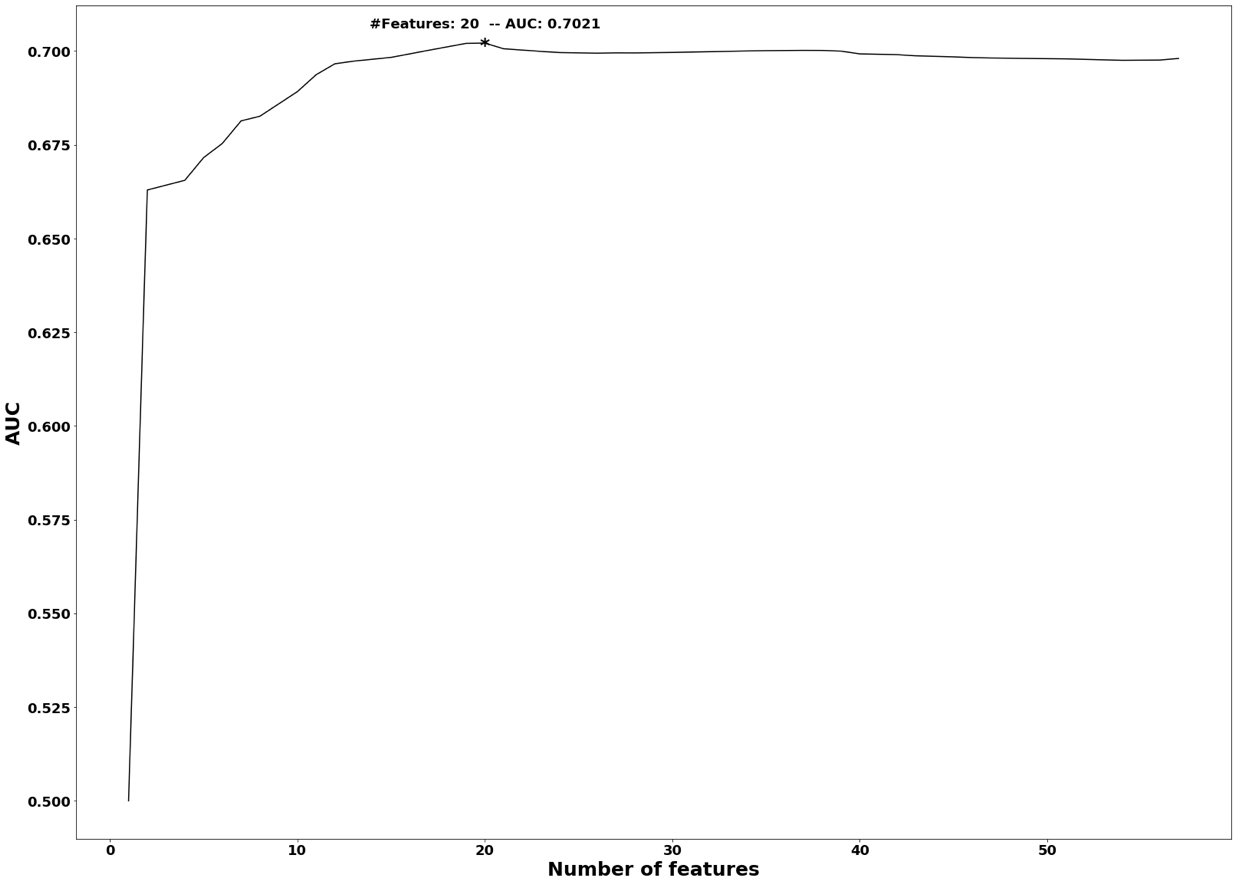


**Fig S1.** Recursive feature elimination for finding the most informative features.

**S3. Feature importance using XGBoost**

Although non-linear models (e.g., RF and XGBoost) are not easy to interpret, their feature importance can provide some insight on the relative importance of each variable. Fig S2 presents the feature importance of the XGBoost model.


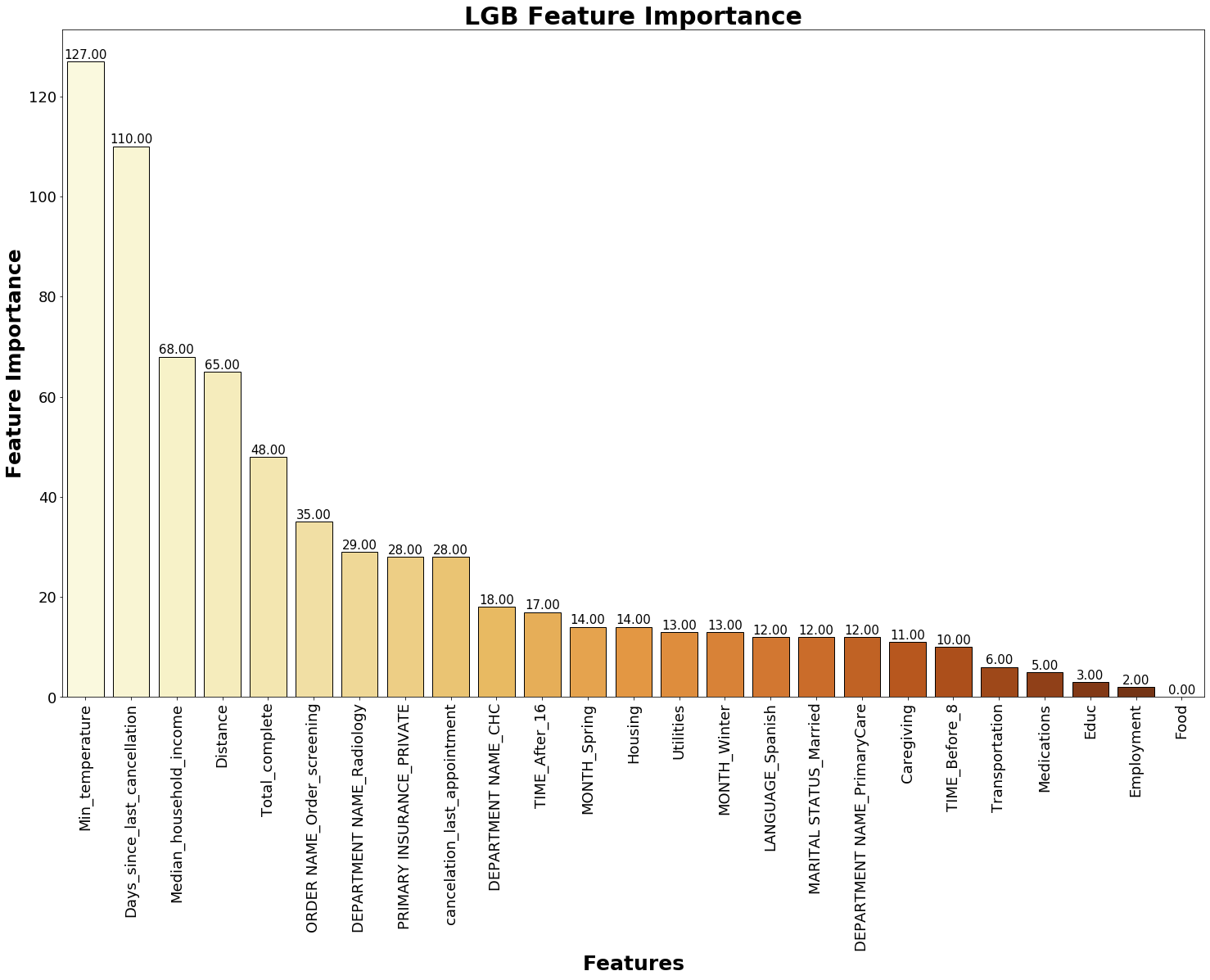


**Fig S2.** The feature importance of the XGBoost model.
